# Higher in-hospital mortality in SARS-CoV-2 omicron variant infection compared to influenza infection—Insights from the CORONA Germany study

**DOI:** 10.1371/journal.pone.0292017

**Published:** 2023-09-27

**Authors:** Jannis Dickow, Melanie A. Gunawardene, Stephan Willems, Johannes Feldhege, Peter Wohlmuth, Martin Bachmann, Martin W. Bergmann, Wolfgang Gesierich, Lorenz Nowak, Ulrich-Frank Pape, Ruediger Schreiber, Sebastian Wirtz, Raphael Twerenbold, Sara Sheikhzadeh, Nele Gessler

**Affiliations:** 1 Asklepios Hospital St. Georg, Department of Cardiology and Internal Intensive Care Medicine, Hamburg, Germany; 2 Semmelweis University, Budapest, Hungary; 3 Asklepios Proresearch, Research Institute, Hamburg, Germany; 4 Asklepios Hospital Harburg, Department of Intensive Care and Ventilatory Medicine, Hamburg, Germany; 5 Asklepios Hospital Wandsbek, Department of Internal Medicine – Cardiology and Pneumology, Hamburg, Germany; 6 Asklepios Hospital Altona, Department of Cardiology and Internal Medicine, Hamburg, Germany; 7 Asklepios Hospital Munich-Gauting, Department of Pneumology, Munich, Germany; 8 Asklepios Hospital München-Gauting, Department of Intensive Care and Ventilation Medicine, Munich, Germany; 9 Asklepios Hospital St. Georg, Department of Internal medicine - Gastroenterology, Hamburg, Germany; 10 Asklepios West-Clinic, Department Anesthesiology and Intensive Care Medicine, Hamburg, Germany; 11 Asklepios Hospital Barmbek, Department Anesthesiology, Intensive Care and Emergency Medicine, Hamburg, Germany; 12 University Heart and Vascular Center Hamburg, Hamburg, Germany; 13 Asklepios Hospitals, Hamburg, Germany; Alfaisal University, SAUDI ARABIA

## Abstract

**Background:**

With the emergence of new subvariants, the disease severity of Severe Acute Respiratory Syndrome Coronavirus-2 has attenuated. This study aimed to compare the disease severity in patients hospitalized with omicron variant infection to those with influenza infection.

**Methods:**

We compared data from the multicenter observational, prospective, epidemiological “CORONA Germany” (Clinical Outcome and Risk in hospitalized COVID-19 patients) study on patients infected with Severe Acute Respiratory Syndrome Coronavirus-2 to retrospective data on influenza infection cases from November 2016 to August 2022. Severe Acute Respiratory Syndrome Coronavirus-2 cases were classified as wild-type/delta variant before January 2022, or omicron variant from January 2022 onward. The primary outcome was in-hospital mortality, adjusted for age, gender, and comorbidities.

**Results:**

The study included 35,806 patients from 53 hospitals in Germany, including 4,916 patients (13.7%) with influenza infection, 16,654 patients (46.5%) with wild-type/delta variant infection, and 14,236 patients (39.8%) with omicron variant infection. In-hospital mortality was highest in patients with wild-type/delta variant infection (16.8%), followed by patients with omicron variant infection (8.4%) and patients with influenza infection (4.7%). In the adjusted analysis, higher age was the strongest predictor for in-hospital mortality (age 80 years vs. age 50 years: OR 4.25, 95% CI 3.10–5.83). Both, patients with wild-type/delta variant infection (OR 3.54, 95% CI 3.02–4.15) and patients with omicron variant infection (OR 1.56, 95% CI 1.32–1.84) had a higher risk for in-hospital mortality than patients with influenza infection.

**Conclusion:**

After adjusting for age, gender and comorbidities, patients with wild-type/delta variant infection had the highest risk for in-hospital mortality compared to patients with influenza infection. Even for patients with omicron variant infection, the adjusted risk for in-hospital mortality was higher than for patients with influenza infection. The adjusted risk for in-hospital mortality showed a strong age dependency across all virus types and variants.

**Trial registration number:**

NCT04659187.

## Introduction

As of February 2023, more than 750 million confirmed cases of COVID-19 have been reported to WHO globally, including more than 6.8 million deaths [1]. With the emergence of new subvariants, the disease severity of Severe Acute Respiratory Syndrome Coronavirus-2 (SARS-CoV-2) infection has attenuated and the mortality rate of the omicron subvariant has approximately halved compared to earlier variants [2].

Although disproportionately more patients became infected with the omicron variant than with the wild-type, beta, or delta variants, only a small proportion suffer a severe course requiring hospitalization [2]. While several studies in the early phase of the pandemic have demonstrated a worse clinical course and higher in-hospital mortality for SARS-CoV-2 wild-type infections compared with influenza infections [3–6], studies directly comparing outcomes of omicron variant infections with influenza infections are rare.

Understanding the behavior of SARS-CoV-2 by reviewing past pandemics and comparing SARS-CoV-2 with similar viral species, we can gain insights to better comprehend the virus’s behavior and effectively respond to it [7, 8]. Therefore, the aim of our study was to investigate whether the disease severity of patients hospitalized with SARS-CoV-2 infection and influenza infection is comparable. Specifically, we investigated whether the omicron variant had comparable in-hospital mortality to seasonal influenza.

## Materials and methods

### Study design and data sources

As described before, the “CORONA Germany”—Clinical Outcome and Risk in hospitalized COVID-19 patients—study (ClinicalTrials.gov, NCT04659187) is a multicenter observational, prospective, epidemiological cohort study [9–14]. The study involved about 53 hospitals across Germany, all belonging to the same hospital group. It was fully investigator-initiated with a steering committee, which was responsible for design, execution and conduct of the study. All data collected from the data repository were validated by the networks’ quality management data base. First results of the study have been published previously [9–14].

Data from the “CORONA Germany” study on SARS-CoV-2 infected patients were compared to retrospective data on influenza infection cases. The database contains, among other things, demographic data, as well as coded diagnosis and procedure codes. For data analysis, all hospital cases with International Statistical Classification of Diseases and Related Deaths (ICD) codes J09, J10, and J11 were extracted. The analyzed timeframe was November 2016 to August 2022, in order to include influenza patients prior to the COVID-19 pandemic.

The statistical analyses and interpretation of the data were authorized by all members of the steering committee. The authors attest to the accuracy of the data and of all analyses. The ethics committee of the General Medical Council (Aerztekammer) for the city of Hamburg and the ethics committee of the General Medical Council (Aerztekammer) for the city of Munich approved the study.

### Study participants

The inclusion criteria for the study were hospitalization with influenza infection or SARS-CoV-2 infection. Adult patients age ≥18 with ICD codes for PCR test-proven SARS-CoV-2 infection (U07.1) or influenza infection (J09, J10, J11) were included in the study. Data collection included patient case information such as age, sex, discharge status, admission and discharge dates, and all ICD-coded diagnoses. Survival status after hospitalization was derived from discharge status.

Patients were grouped by A) influenza infection, B) wild-type to delta variant SARS-CoV-2 infection, and C) omicron variant SARS-CoV-2 infection. SARS-CoV-2 cases with discharge dates before January 2022 were classified as wild-type or delta variant infection and those with discharge dates from January 2022 onward were classified as omicron variant infection. This classification was based on the predominant SARS-CoV-2 variant in Germany according to the Robert Koch Institute [15]. In case of dual coding of influenza infection and SARS-CoV-2 infection, patients were assigned to SARS-CoV-2 infection.

### Statistical methods

#### Outcomes

The primary outcome of this retrospective analysis was in-hospital mortality during influenza infection or SARS-CoV-2 infection (wild-type to delta variant or omicron variant). Secondary outcomes were pneumonia, ventilation and length of hospital stay.

#### Descriptive analyses

Patient demographics and comorbidities were compared between viral types. All data were analyzed descriptively and are presented in summary tables. Metric data are shown as means +/- standard deviations and as medians [25th and 75th percentiles]. Categorical data are presented as frequencies and proportions. The analyses were stratified by virus type and virus variant.

#### Comorbidities

In addition to the viral variant, a variety of patient characteristics could influence mortality, some of which were also distributed differently between viral variants. Therefore, analyses were adjusted for the following patient characteristics and comorbidities classified by ICD-codes: Age, gender, tumor disease (C00*—C97*), diabetes mellitus (E10*—E14*), lipid metabolism disorder (E78*), obesity (E66*), heart failure (I50*), ischemic heart disease (I20*—I25*), cerebrovascular disease (I60*—I69*), liver cirrhosis/fibrosis (K70.3* and K74*), chronic obstructive pulmonary disease (COPD, J44).

#### Regression models

A logistic regression model was applied to associate the virus type of the patients with survival. The analyses were adjusted for age, gender, and comorbidities (tumor disease, diabetes mellitus, lipid metabolism disorder, obesity, heart failure, ischemic heart disease, cerebrovascular disease, liver cirrhosis/fibrosis, COPD). An interaction of virus type with age was included in the model. Missing data were imputed using additive regression models. Odds ratios and 95% confidence intervals were shown. All p-values were two-sided and a p-value <0.05 was considered significant. All calculations were performed with the statistical analysis software R (R Core Team, 2022). Details are shown in S1–S5 Tables.

## Results

### Baseline characteristics

From November 2016 to August 2022, 35,806 patients out of 53 hospitals in Germany were analyzed. Of these patients, 4,916 patients (13.7%) were hospitalized with influenza infection and 30,890 patients (86.3%) with SARS-CoV-2 infection. Ten patients (0.03%) were diagnosed with both SARS-CoV-2 infection and influenza infection on admission and were classified as SARS-CoV-2 infection. In 2020 and 2021, 16,654 patients (46.5%) were admitted with SARS-CoV-2 infection and classified as wild-type to delta variant infection; in 2022, 14,236 patients (39.8%) were admitted with SARS-CoV-2 infection and classified as omicron variant infection.

Baseline characteristics and comorbidity rates are presented in Tables 1 and 2. The gender distribution was balanced across all groups (female gender: influenza 49.9%, wild-type to delta 47.5%, omicron 50.2%). Age was highest in the omicron group at median 72 [IQR 53–83] years compared to 68 [IQR 47–80] years in the influenza group and 70 [IQR 54–82] years in the wild-type to delta group.

**Table 1:** Baseline data stratified by virus type and variant.

	Overall	Influenza	Wild-type/Delta	Omicron
	N = 35,806	N = 4,916	N = 16,654	N = 14,236
Gender				
• male	51.0 (18,259)	50.1 (2,463)	52.4 (8,727)	49.7 (7,069)
• female	48.9 (17,512)	49.9 (2,452)	47.5 (7,911)	50.2 (7,149)
• missing	35	1	16	18
Age (years)				
• Median [Q1, Q3]	71 [53, 82]	68 [47, 80]	70 [54, 82]	72 [53, 83]
• Mean (SD)	65 (22)	60 (25)	66 (19)	65 (23)

Metric data are shown as means (standard deviations) and as medians [25th and 75th percentiles]. Categorical data are presented as percentages (absolute numbers). The analyses were stratified by virus type and virus variant.

**Table 2:** Comorbidities stratified by virus type and variant.

	Overall	Influenza	Wild-type/Delta	Omicron
	N = 35,806	N = 4,916	N = 16,654	N = 14,236
Tumor disease	5.3 (1,891)	3.8 (189)	4.7 (781)	6.5 (921)
Diabetes mellitus	22.0 (7,862)	18.8 (923)	24.8 (4,131)	19.7 (2,808)
Lipid metabolic disorder	15.4 (5,528)	14.6 (720)	15.7 (2,620)	15.4 (2,188)
Obesity	5.1 (1,822)	4.9 (241)	6.4 (1,070)	3.6 (511)
Heart Failure	15.4 (5,210)	15.0 (738)	14.2 (2,371)	14.8 (2,101)
Ischemic heart disease	14.1 (5,053)	15.4 (759)	13.9 (2,318)	13.9 (1,976)
Cerebrovascular disease	7.5 (2,683)	6.6 (322)	7.4 (1,226)	8.0 (1,135)
Liver cirrhosis/fibrosis	0.9 (325)	0.8 (39)	0.8 (130)	1.1 (156)
COPD	7.3 (2,619)	12.0 (588)	6.4 (1,061)	6.8 (970)

Categorical data are presented as frequencies and absolute numbers. The analyses were stratified by virus type and virus variant. COPD indicates chronic obstructive pulmonary disease.

The rates of patients with lipid metabolic disorder (influenza 14.6%, wild-type to delta 15.7%, omicron 15.4%), heart failure (influenza 15.0%, wild-type to delta 14.2%, omicron 14.8%), and liver cirrhosis/fibrosis (influenza 0.8%, wild-type to delta 0.8%, omicron 1.1%) were similar among groups. The rates of patients with cerebrovascular disease (influenza 6.6%, wild-type to delta 7.4%, omicron 8.0%) or tumor disease (influenza 3.8%, wild-type to delta 4.7%, omicron 6.5%) were highest in patients with omicron variant infection. The relative frequencies of patients with diabetes mellitus (influenza 18.8%, wild-type to delta 24.8%, omicron 19.7%) and obesity (influenza 4.9%, wild-type to delta 6.4%, omicron 3.6%) were highest in patients with wild-type to delta variant infection. The only comorbidities most frequently found in patients with influenza infection were ischemic heart disease (influenza 15.4%, wild-type to delta 13.9%, omicron 13.9%) and COPD (influenza 12.0%, wild-type to delta 6.4%, omicron 6.8%).

### Primary and secondary outcomes

The rates of pneumonia were 55.5% (n = 9,235/16,654) in the wild-type to delta group compared to 28.7% (n = 1,412/4,916) in the influenza group and 18.4% (n = 2,619/14,236) in the omicron group (Table 3). In the wild-type to delta group, 14.9% of patients (n = 2,489/16,654) required ventilation compared to 7.6% of patients (n = 375/4,916) in the influenza group and 4.9% of patients (n = 703/14,236) in the omicron group (Table 3). The risk of requiring ventilation was not different between patients with omicron variant infection compared with influenza infection (OR 0.87 [95% CI 0.64–1.17]). However, with wild-type to delta variant infection, the odds ratio was 3.66 (95% CI 2.78–4.82) compared with influenza infection and 4.21 (95% CI 3.61–4.90) compared with omicron infection (Table 3).

**Table 3:** Primary and secondary outcomes stratified by virus type and variant.

Outcomes	Overall	Influenza	Wild-type/Delta	Omicron
	N = 35,806	N = 4,916	N = 16,654	N = 14,236
Pneumonia	37.0 (13,266)	28.7 (1,412)	55.5 (9,235)	18.4 (2,619)
Ventilation	10.0 (3,567)	7.6 (375)	14.9 (2,489)	4.9 (703)
In-hospital mortality	11.7 (4,214)	4.7 (231)	16.8 (2,791)	8.4 (1,192)
Length of stay (days)				
• Median [Q1, Q3]	7 [3, 16]	6 [3, 11]	8 [4, 17]	7 [3, 15]
• Mean (SD)	13 (19)	11 (17)	15 (22)	12 (17)

Metric data are shown as means (standard deviations) and as medians [25th and 75th percentiles]. Categorical data are presented as frequencies and absolute numbers. The analyses were stratified by virus type and virus variant.

Overall, 11.8% of patients (n = 4,214/35,806) died in the hospital during the course of the infection. In-hospital mortality was highest in patients with wild-type to delta variant SARS-CoV-2 infection (16.8%, n = 2,791) followed by patients with omicron variant SARS-CoV-2 infection (8.4%, n = 1,192). The lowest rate of in-hospital mortality was found in patients with influenza infection (4.7%, n = 231; Table 3).

### Adjusted analyses of in-hospital mortality

When adjusted for age, gender, and comorbidities (tumor disease, diabetes mellitus, lipid metabolism disorder, obesity, heart failure, ischemic heart disease, cerebrovascular disease, liver cirrhosis/fibrosis, COPD), strong predictors for in-hospital mortality were age (80 years compared to 50 years: OR 4.25 [95% CI 3.10–5.83]), liver cirrhosis/fibrosis (OR 2.63 [95% CI 1.96–3.51]) and tumor disease (OR 2.24 [95% CI 1.98–2.53]).

**Fig 1:**
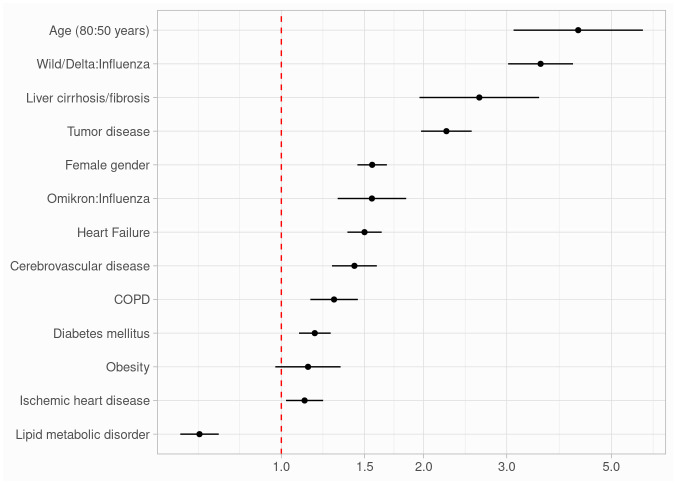
Forest plot of model effects. When adjusted for age, gender, and comorbidities, age, liver cirrhosis/fibrosis and tumor disease had a strong impact on in-hospital mortality. Among virus groups, patients with wild-type to delta variant infection had a higher risk for in-hospital mortality compared to patients with influenza infection. Furthermore, patients with omicron variant infection had a higher risk for in-hospital mortality when compared to patients with influenza infection. COPD indicates chronic obstructive pulmonary disease.

Among virus groups, patients with wild-type to delta variant infection had a higher risk for in-hospital mortality compared to patients with influenza infection (OR 3.54 [95% CI 3.02–4.15]). Furthermore, patients with omicron variant infection had a higher risk for in-hospital mortality when compared to patients with influenza infection (OR 1.56 [95% CI 1.32–1.84]). Model effects from the adjusted logistic regression model are presented in Table 4 and Fig 1.

**Table 4:** Model effects from the adjusted logistic regression model.

Term	Odds ratio	95% confidence interval
Age (80:50 years)	4.25	3.10–5.83
Female gender	1.56	1.45–1.67
Wild/Delta:Influenza	3.54	3.02–4.15
Omicron:Influenza	1.56	1.32–1.84
Tumor disease	2.24	1.98–2.53
Diabetes mellitus	1.18	1.09–1.27
Lipid metabolic disorder	0.67	0.61–0.74
Obesity	1.14	0.97–1.34
Heart Failure	1.50	1.38–1.63
Ischemic heart disease	1.12	1.02–1.23
Cerebrovascular disease	1.43	1.28–1.59
Liver cirrhosis/fibrosis	2.63	1.96–3.51
COPD	1.29	1.15–1.45

Compared to influenza infection, the omicron variant had a 1.56-fold odds and the wild-type to delta variant a 3.54-fold odds of mortality. The odds ratios above were adjusted for a median age of 71 years and the influenza group.

**Fig 2:**
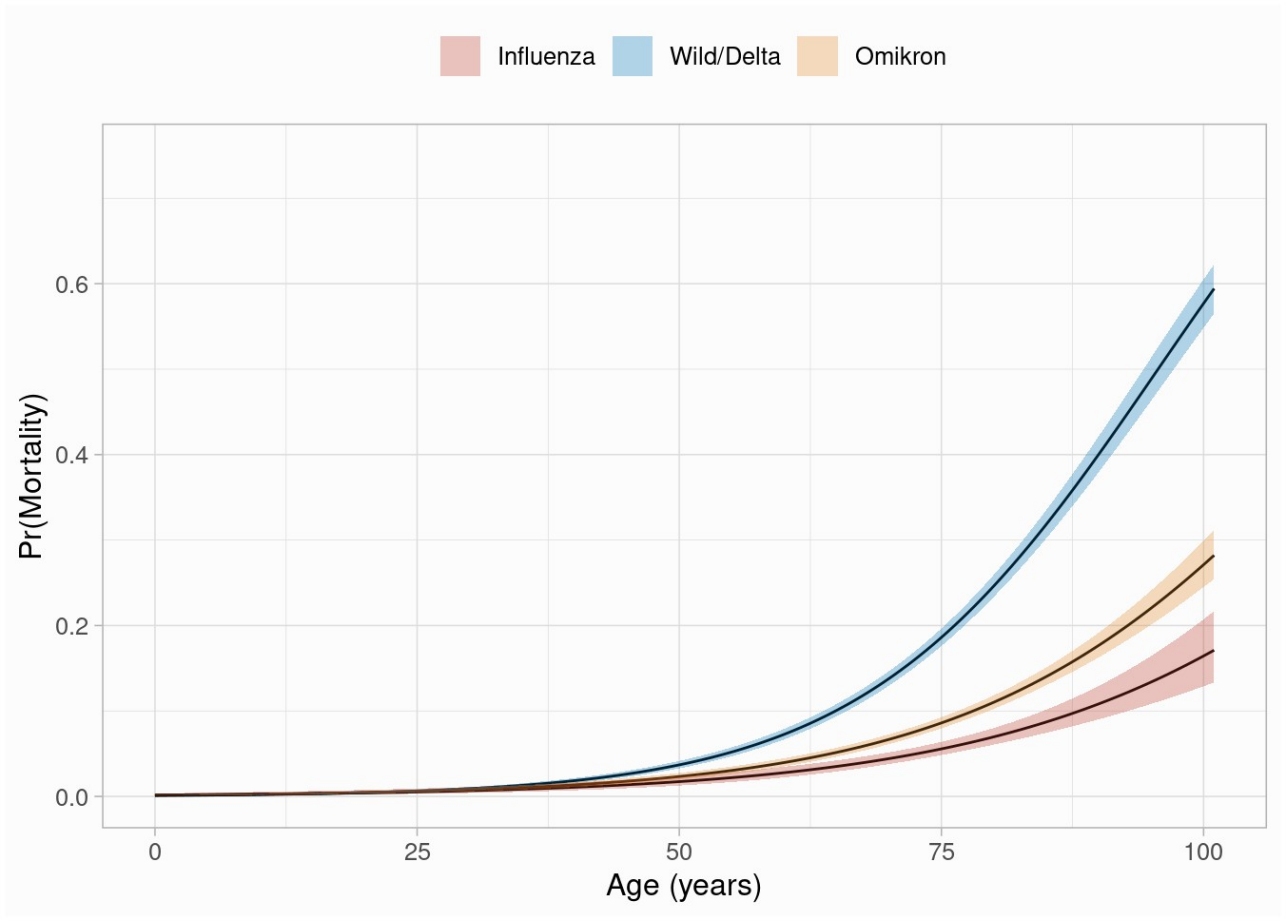
Model-based probabilities of mortality by virus type and variant. Wild-type to delta infection increased the risk for in-hospital mortality compared to influenza infection or omicron variant infection. Infection with the omicron variant was also associated with higher mortality, but the difference to influenza infection remains moderate.

Odds ratios for the virus type / variants based on the regression model of mortality change with increasing years of patient life are shown in Table 5. Comparing 30-, 50- and 70-year-olds, the odds of dying from omicron variant infection compared with influenza infection are 1.18-, 1.35- and 1.55-fold. The odds of mortality from wild-type to delta variant compared with influenza infection are 1.37-, 2.17- and 3.46-fold, and from infection with wild-type or delta variant compared with omicron variant are 1.16-, 1.61-, and 2.24-fold. These results are also shown in Fig 2, which shows model-based probabilities of mortality by virus type and variant in relation to age.

**Table 5:** Model-based in-hospital mortality probabilities by virus types and variants at different ages.

Age	Omicron vs Influenza		Wild-type/Delta vs Influenza		Wild-type/Delta vs Omicron	
	Odds ratio	95% confidence interval	Odds ratio	95% confidence interval	Odds ratio	95% confidence interval
30	1.18	0.66–2.09	1.37	0.79–2.36	1.16	0.84–1.60
35	1.22	0.73–2.05	1.54	0.94–2.51	1.26	0.94–1.69
40	1.26	0.80–2.00	1.72	1.11–2.67	1.37	1.05–1.77
45	1.31	0.87–1.97	1.94	1.32–2.85	1.48	1.18–1.87
50	1.35	0.95–1.92	2.17	1.56–3.04	1.61	1.32–1.97
55	1.40	1.03–1.89	2.44	1.84–3.25	1.75	1.47–2.08
60	1.44	1.12–1.86	2.74	2.16–3.48	1.90	1.65–2.19
65	1.49	1.21–1.84	3.08	2.53–3.75	2.06	1.83–2.32
70	1.55	1.30–1.84	3.46	2.94–4.07	2.24	2.03–2.46

After adjusting for comorbidities, the model-based risk for in-hospital mortality differed between virus types and variants and increased with age for both, omicron variant infection and wild-type to delta infection when compared to influenza infection.

## Discussion

Key findings of this prospective observational multicenter study are: First, patients admitted for SARS-CoV-2 infection had a higher risk for in-hospital mortality compared with patients admitted for influenza infection. After adjusting for age and several relevant comorbidities, a 1.56-fold odds for in-hospital mortality was observed for patients with omicron variant infection, and a 3.54-fold odds for patients with wild-type to delta variant infection, respectively. Second, increasing age was a strong predictor for in-hospital mortality.

Our results are in line with previous large studies comparing the disease severity in terms of in-hospital outcomes of influenza infection and SARS-CoV-2 infection in the beginning of the pandemic when wild-type to delta variant infections were predominant, and the age-dependency of severe outcomes and in-hospital mortality are supported by several analyses [3, 5, 16–18]. In a Japanese cohort study using administrative claims data in Japan, patients admitted to the hospital for SARS-CoV-2 infection had an approximately two-fold higher risk to die in the hospital than patients admitted for influenza infection [16]. Stratified by age, this effect only remained for patients older than 70 years of age [16]. In a retrospective study among hospitalized US veterans older than 65 years of age, 30-day mortality from SARS-CoV-2 infection exceeded that from influenza infection with 18.9% compared to 4.3% [17]. Further German healthcare claims data showed, severe outcomes occurred in one-third of patients with SARS-CoV-2 infection, but only in one-sixth of patients with influenza infection [18]. For both groups, the risk of intensive care, mechanical ventilation, and in-hospital mortality increased with age but was overall higher in patients with SARS-CoV-2 infection [18].

Studies on the comparison of outcomes among patients hospitalized with the omicron variant and those hospitalized with influenza infection are rare. In unvaccinated children, infection with the omicron variant was associated with a higher risk for intensive care, mechanical ventilation, and oxygen use than infection with influenza [19]. In line with the findings of our study, in the first prospective multicenter cohort study from Switzerland, SARS-CoV-2 omicron variant infection was associated with a 1.54-fold higher risk of in-hospital mortality compared with influenza infection [20]. Furthermore, older age and dementia were associated with a higher risk for in-hospital mortality before intensive care unit admission [20].

The observed rates of comorbidities in patients admitted to the hospital for influenza infection differed to the comorbidities of patients with SARS-CoV-2 infection supporting prior population-level data, which demonstrated, that diabetes and obesity are risk factors for hospital admission in patients with SARS-CoV-2 infection [5, 21–23]. For both, rates were higher in patients with omicron variant infection compared to patients with influenza infection. Whereas in patients with influenza infection, ischemic heart disease and COPD were more frequent. Interestingly, however, after adjusting for these comorbidities, in-hospital mortality was still higher in patients with omicron variant infection.

Of note, the rates of pneumonia in patients with influenza infection were higher than in patients with omicron variant infection. Likely because the omicron variant causes more disease in the upper airway compared to influenza [19], which could be explained by the fact, that omicron replicates faster in the bronchi but less efficiently in the lung parenchyma as observed in ex vivo explant cultures [24]. Furthermore, patients with SARS-CoV-2 infection admitted to the hospital are at increased risk of systemic clinical manifestations and extrapulmonary organ dysfunction such as acute kidney injury, severe septic shock, arrythmias, sudden cardiac death, and elevated troponin [6, 10]. However, when adjusted for age, gender, and comorbidities such as COPD, the rates risk of requiring mechanical ventilation did not differ between patients with influenza infection and patients with omicron variant infection.

Comparing the behavior and characteristics of SARS-CoV-2 with other virus species plays a critical role in identifying patterns, predicting future behavior, and developing targeted strategies to understand the past pandemic and combat future virus outbreaks. Comparative analysis of these viruses enhances our ability to understand, control, and respond effectively to the challenges posed by SARS-CoV-2 and other virus species [7, 8].

## Limitations

Several limitations need to be mentioned. Although this study shows an unselected, real world patient population out of 53 hospitals, our results may not represent the entire population in Germany. Inclusion was based only on ICD codes for PCR test-proven SARS-CoV-2 or influenza infection. Therefore, patients at all stages of the disease were included as well as patients who were admitted for other reasons and had a positive PCR tests concomitant. Information on vaccination status was not available for either virus types and could not be considered for inclusion. Because of the classification of SARS-CoV-2 variant by time of hospital admission and predominant variant, it is likely that some patients in the study were misclassified during the transition period. Furthermore, different time episodes were compared for each virus type and variant which could lead to the inclusion of unknown confounders for in-hospital mortality. Administrative data can be subject to misclassification although billing codes are monitored by payors and hospitals during the reimbursement process. Lastly, causes of death were unknown.

## Conclusion

In this large prospective observational cohort study, patients admitted to the hospital for SARS-CoV-2 infection had higher rates of in-hospital mortality than patients with influenza infection. After adjusting for age and other relevant comorbidities, patients with wild-type to delta variant infection had the highest risk for in-hospital mortality with a more than three-fold increased risk compared to patients with influenza infection. Even for patients with omicron variant infection the adjusted risk for in-hospital mortality was almost two-fold higher than for patients with influenza infection. The adjusted risk for in-hospital mortality showed a strong age dependency in all virus types and variants.

## Supporting information

S1 Table Data dictionary. The processed data, labels, variable type and values.
(DOCX)

S2 Table Definition of virus variants. (DOCX)

S3 Table Used R packages. (DOCX)

S4 Table Model summary: Prediction of mortality. (DOCX)

S5 Table Model summary: Prediction of ventilation and effect sizes. (DOCX)
