# Supplementary material for: Higher in-hospital mortality in SARS-CoV-2 omicron variant infection compared to influenza infection—Insights from the CORONA Germany study
Source: PLoS One. 2023 Sep 27;18(9):e0292017. doi: 10.1371/journal.pone.0292017 (PMC10529565; doi:10.1371/journal.pone.0292017)
Supplement: S1 Table — The processed data, labels, variable type and values. (DOCX) [file pone.0292017.s001.docx]

## S.1 Table: Data dictionary

The processed data, labels, variable type and values.

| **Variable name** | **Description** | **Type** | **Values** |
| --- | --- | --- | --- |
| F_sex | Gender | factor | "male", "female" |
| F_age | Age (years) | integer | 0 - 104 |
| F_tod | Mortality | factor | "no", "yes" |
| F_beatmung | Ventilation | factor | "no", "yes" |
| D_tumor | Tumor disease (C00-C97) | factor | "no", "yes" |
| D_dm | Diabetes mellitus (E10 – E14) | factor | "no", "yes" |
| D_lipid | Lipid metabolic disorder (E78) | factor | "no", "yes" |
| D_adipositas | Obesity (E66) | factor | "no", "yes" |
| D_hi | Heart Failure (I50) | factor | "no", "yes" |
| D_ischhk | Ischemic heart disease (I20 – I25) | factor | "no", "yes" |
| D_cerebrov | Cerebrovascular disease (I60 – I69) | factor | "no", "yes" |
| D_leberzirrh | Liver cirrhosis/fibrosis (K70.3 und K74) | factor | "no", "yes" |
| D_cpd | Chronic pulmonary disease (J44, J45, E84, J84.1) | factor | "no", "yes" |
| D_J44 | COPD (J44) | factor | "no", "yes" |
| D_J45 | Asthma (J45) | factor | "no", "yes" |
| D_J84.1 | Pulmonary fibrosis (J84.1) | factor | "no", "yes" |
| gruppe | Diagnosegruppe | factor | "Influenza", "Wild/Delta", "Omikron" |
| mb | Mechanical Ventilation (OPS 8-711 - 8-713) | factor | "no", "yes" |
