# Supplementary material for: Higher in-hospital mortality in SARS-CoV-2 omicron variant infection compared to influenza infection—Insights from the CORONA Germany study
Source: PLoS One. 2023 Sep 27;18(9):e0292017. doi: 10.1371/journal.pone.0292017 (PMC10529565; doi:10.1371/journal.pone.0292017)
Supplement: S2 Table — (DOCX) [file pone.0292017.s002.docx]

## S.2 Table: Definition of virus variants

| **Virus** | **ICD-Classification** | **Temporal delimination** |
| --- | --- | --- |
| Influenza | J09, J10, J11 |  |
| Wild-type/Delta variant | U07.1 | before January 2022 |
| Omikron | U07.1 | since January 2022 |
