# Supplementary material for: Higher in-hospital mortality in SARS-CoV-2 omicron variant infection compared to influenza infection—Insights from the CORONA Germany study
Source: PLoS One. 2023 Sep 27;18(9):e0292017. doi: 10.1371/journal.pone.0292017 (PMC10529565; doi:10.1371/journal.pone.0292017)
Supplement: S3 Table — (DOCX) [file pone.0292017.s003.docx]

## S.3 Table: Used R packages

| **R Package** | **Version** | **Date** | **R Version** | **Task** |
| --- | --- | --- | --- | --- |
| DBI | 1.1.3 | 2022-06-18 | R 4.2.0 | Data import |
| odbc | 1.3.3 | 2021-11-30 | R 4.2.0 | Data import |
| readr | 2.1.2 | 2022-01-30 | R 4.2.0 | Data import |
| readxl | 1.4.0 | 2022-03-28 | R 4.2.0 | Data import |
| dplyr | 1.0.9 | 2022-04-28 | R 4.2.0 | Data preparation |
| forcats | 0.5.1 | 2021-01-27 | R 4.2.0 | Data preparation |
| Hmisc | 4.7-0 | 2022-04-19 | R 4.2.0 | Data preparation |
| lubridate | 1.8.0 | 2021-10-07 | R 4.2.0 | Data preparation |
| purrr | 0.3.4 | 2020-04-17 | R 4.2.0 | Data preparation |
| stringr | 1.4.0 | 2019-02-10 | R 4.2.0 | Data preparation |
| tibble | 3.1.7 | 2022-05-03 | R 4.2.0 | Data preparation |
| tidyr | 1.2.0 | 2022-02-01 | R 4.2.0 | Data preparation |
| tidyverse | 1.3.1 | 2021-04-15 | R 4.2.0 | Data preparation |
| gtsummary | 1.6.1 | 2022-06-22 | R 4.2.0 | Descriptives |
| ggplot2 | 3.4.0 | 2022-11-04 | R 4.2.0 | Graphics |
| ggsci | 2.9 | 2018-05-14 | R 4.2.0 | Graphics |
| scales | 1.2.0 | 2022-04-13 | R 4.2.0 | Graphics |
| emmeans | 1.7.5 | 2022-06-22 | R 4.2.0 | Modelling |
| rms | 6.3-0 | 2022-04-22 | R 4.2.0 | Modelling |
