# Supplementary material for: Higher in-hospital mortality in SARS-CoV-2 omicron variant infection compared to influenza infection—Insights from the CORONA Germany study
Source: PLoS One. 2023 Sep 27;18(9):e0292017. doi: 10.1371/journal.pone.0292017 (PMC10529565; doi:10.1371/journal.pone.0292017)
Supplement: S4 Table — (DOCX) [file pone.0292017.s004.docx]

## S.4 Table: Model summary: prediction of mortality

| **term** | **estimate** | **std.error** | **statistic** | **p.value** |
| --- | --- | --- | --- | --- |
| Intercept | -6.450 | 0.418 | -15.430 | < 0.001 |
| F_sex=female | -0.443 | 0.037 | -12.106 | < 0.001 |
| gruppe=Wild/Delta | -0.382 | 0.445 | -0.860 | 0.390 |
| gruppe=Omikron | -0.037 | 0.468 | -0.079 | 0.937 |
| F_age | 0.048 | 0.005 | 8.900 | < 0.001 |
| D_tumor=yes | 0.805 | 0.063 | 12.798 | < 0.001 |
| D_dm=yes | 0.163 | 0.039 | 4.140 | < 0.001 |
| D_lipid=yes | -0.400 | 0.048 | -8.341 | < 0.001 |
| D_adipositas=yes | 0.130 | 0.081 | 1.603 | 0.109 |
| D_hi=yes | 0.405 | 0.043 | 9.509 | < 0.001 |
| D_ischhk=yes | 0.114 | 0.046 | 2.460 | 0.014 |
| D_cerebrov=yes | 0.356 | 0.056 | 6.383 | < 0.001 |
| D_leberzirrh=yes | 0.965 | 0.149 | 6.491 | < 0.001 |
| D_J44=yes | 0.257 | 0.059 | 4.348 | < 0.001 |
| gruppe=Wild/Delta * F_age | 0.023 | 0.006 | 4.075 | < 0.001 |
| gruppe=Omikron * F_age | 0.007 | 0.006 | 1.132 | 0.258 |
