# Supplementary material for: Higher in-hospital mortality in SARS-CoV-2 omicron variant infection compared to influenza infection—Insights from the CORONA Germany study
Source: PLoS One. 2023 Sep 27;18(9):e0292017. doi: 10.1371/journal.pone.0292017 (PMC10529565; doi:10.1371/journal.pone.0292017)
Supplement: S5 Table — (DOCX) [file pone.0292017.s005.docx]

# S.5 Table: Model summary: prediction of ventilation and effect sizes

| **term** | **estimate** | **std.error** | **statistic** | **p.value** |
| --- | --- | --- | --- | --- |
| Intercept | -4.009 | 0.136 | -29.465 | < 0.001 |
| gruppe=Wild/Delta | 1.297 | 0.140 | 9.270 | < 0.001 |
| gruppe=Omikron | -0.140 | 0.153 | -0.910 | 0.363 |

| **term** | **Odds Ratio** | **95% confidence interval** |
| --- | --- | --- |
| Influenza:Omikron | 1.149 | 0.852 - 1.551 |
| Wild/Delta:Omikron | 4.207 | 3.615 - 4.896 |
